# TGFβR-1/ALK5 inhibitor RepSox induces enteric glia-to-neuron transition and influences gastrointestinal mobility in adult mice

**DOI:** 10.1038/s41401-022-00932-4

**Published:** 2022-07-06

**Authors:** Chang-jie Shi, Jun-jiang Lian, Bo-wen Zhang, Jia-xue Cha, Qiu-hong Hua, Xiao-ping Pi, Yu-jun Hou, Xin Xie, Ru Zhang

**Affiliations:** 1 Shanghai Key Laboratory of Signaling and Disease Research, Laboratory of Receptor-based Bio-medicine, School of Life Sciences and Technology, Tongji University, Shanghai, 200092 China; 2 CAS Key Laboratory of Receptor Research, the National Center for Drug Screening, Shanghai Institute of Materia Medica, Chinese Academy of Sciences, Shanghai, 201203 China

**Keywords:** enteric glial cells, enteric neuropathies, TGFβR-1/ALK5, VCR, RepSox, gastroenterology

## Abstract

Promoting adult neurogenesis in the enteric nervous system (ENS) may be a potential therapeutic approach to cure enteric neuropathies. Enteric glial cells (EGCs) are the most abundant glial cells in the ENS. Accumulating evidence suggests that EGCs can be a complementary source to supply new neurons during adult neurogenesis in the ENS. In the brain, astrocytes have been intensively studied for their neuronal conversion properties, and small molecules have been successfully used to induce the astrocyte-to-neuron transition. However, research on glia-to-neuron conversion in the ENS is still lacking. In this study, we used GFAP-Cre:Rosa-tdTomato mice to trace glia-to-neuron transdifferentiation in the ENS in vivo and in vitro. We showed that GFAP promoter-driven tdTomato exclusively labelled EGCs and was a suitable marker to trace EGCs and their progeny cells in the ENS of adult mice. Interestingly, we discovered that RepSox or other ALK5 inhibitors alone induced efficient transdifferentiation of EGCs into neurons in vitro. Knockdown of ALK5 further confirmed that the TGFβR-1/ALK5 signalling pathway played an essential role in the transition of EGCs to neurons. RepSox-induced neurons were Calbindin- and nNOS-positive and displayed typical neuronal electrophysiological properties. Finally, we showed that administration of RepSox (3, 10 mg· kg^−1^ ·d^−1^, i.g.) for 2 weeks significantly promoted the conversion of EGCs to neurons in the ENS and influenced gastrointestinal motility in adult mice. This study provides a method for efficiently converting adult mouse EGCs into neurons by small-molecule compounds, which might be a promising therapeutic strategy for gastrointestinal neuropathy.

## Introduction

The enteric nervous system (ENS) is composed of the myenteric plexus and submucosal plexus in the gut wall. This system mainly contains enteric glial cells (EGCs) and enteric neurons [1]. In addition, the ENS is often referred to as ‘the second brain’ because of its complexity and importance in regulating gastrointestinal motility [2]. A variety of adverse factors, such as inflammation, infection, radiation, chemical toxins, surgery, ageing, dietary changes, hyperglycaemia, and metabolic disorders, may lead to neuronal damage or loss of ENS, thereby causing gastrointestinal motility and sensory dysfunction, such as gastroparesis, intractable or slow transit constipation, irritable bowel syndrome and Hirschsprung’s disease [3–9]. However, the means to produce new neurons in the adult gut is somewhat limited. For children with Hirschsprung’s disease, the surgical removal of the entire colon or part of the colon is still the primary choice of treatment. However, such treatment cannot prevent life-long complications such as enterocolitis, constipation, or incontinence occurring later in life. Therefore, addition of new enteric neurons to replace damaged neurons will be a potential solution for repairing ENS dysfunction.

Somatic cell reprogramming and transdifferentiation technologies provide new strategies for modelling neurological diseases [10]. Recent studies have found that epigenetic reprogramming either by forced expression of transcription factors or treatment with chemical compounds can drive the conversion of brain astrocytes into neuronal cells [11–14]. EGCs in the gut resemble CNS astrocytes and are the most abundant cell type in the ENS, which have the same origin as enteric neurons [15]. In addition to morphological characteristics, EGCs share many other features with brain astrocytes, such as the expression of the glial markers GFAP and S100β [16–20], and provide similar functional support to their interconnected neurons [21]. In rodents, enteric neurogenesis is mainly restricted to embryonic stages and early postnatal life. However, a few recent reports have provided compelling evidence for enteric neurogenesis in adult animals in response to 5-hydroxytryptamine 4 (5-HT_4_) receptor activation or by tissue dissociation or injury [22–26]. Even some limited neurogenesis occurs under physiological conditions in the adult ENS [27]. EGCs were suggested to be the precursors of these neurons, but the mechanisms initiating EGC-neuron conversion are still unclear. Moreover, glial cells do not constitutively replace neurons, and neurogenesis in the ENS is not easily provoked. Therefore, it is worth exploring strategies to promote EGC-neuron conversion, especially the endogenous neurogenic potential of enteric glia, which might be harnessed therapeutically to prevent pathological neuron loss in the ENS.

Small chemical molecules are easy to handle and have previously been successfully applied to replace transcription factors in astrocyte-neuron conversion processes in the brain [11–14]. The chemical cocktail VCR (valproic acid, Chir99021, and RepSox) was reported to convert mouse brain astrocytes directly into neuronal cells. Here, we show that RepSox, a small-molecule inhibitor of TGFβR-1/ALK5, induces EGCs to produce neurons in vivo and in vitro. Our results might provide a feasible strategy for in situ regenerative compensation and repair of damaged gastrointestinal neurons.

## Materials and methods

### Animals

GFAP-Cre mice (Jackson Laboratory, Bar Harbour, ME, USA, stock number J012886) express Cre recombinase under the control of the mouse glial fibrillary acidic protein (GFAP) promoter. Rosa26-tdTomato mice (Jackson Laboratory, stock number J007905) express robust tdTomato fluorescence following Cre-mediated recombination. The GFAP-Cre:Rosa26-tdTomato mice used in the experiments were obtained by mating GFAP-Cre male and Rosa26-tdTomato female mice. EGCs derived from such mice were labelled with tdTomato fluorescence, which traced EGCs. All mice were housed in the pathogen-free environment of the Experimental Animal Centre of Tongji University, Shanghai, China. The experimental procedures were performed in accordance with the Tongji University Guide for the Use of Laboratory Animals.

### Isolation and culture of EGCs from the myenteric plexus of adult GFAP-Cre:Rosa26-tdTomato mice

Adult GFAP-Cre:Rosa26-tdTomato mice were anaesthetised by injecting tribromoethanol and decapitated. The ileum was removed and placed in ice-cold Hank’s Buffered Salt Solution (HBSS). The longitudinal muscle myenteric plexus (LMMP) was isolated and rinsed twice with HBSS. LMMP was cut with scissors and digested in 1.3 mg/mL collagenase-type II for 90 min at 37 °C. The disintegrated tissue was centrifuged, followed by incubation in 0.25% trypsin for 13 min at 37 °C. After trypsinization was blocked with DMEM containing 10% FBS, the cells were collected by centrifugation (310 × *g* for 5 min). The cells were resuspended in glial cell medium consisting of DMEM/F12 (Gibco), 10% foetal bovine serum (Gibco), B-27 (Gibco), 20 ng/mL epidermal growth factor (EGF, Invitrogen), 20 ng/mL basic fibroblast growth factor (bFGF, Invitrogen), and penicillin/streptomycin (Gibco) and cultured in a humidified atmosphere of 95% air/5% CO_2_ at 37 °C. Half of the glial cell media was changed every 2–3 days. These isolated cells were cultured for 9 days and defined as the P0 generation. Then, the cells were collected by trypsinization (0.05% trypsin for 11 min) and preserved in liquid nitrogen. Frozen cells were recovered and defined as the P1 generation.

### Immunofluorescence analysis of ileal tissue and cultured cells

Adult GFAP-Cre:Rosa26-tdTomato mice were anaesthetised and decapitated. The ileal segments and LMMP were removed and then fixed in 4% paraformaldehyde for 2 h. For the ileal segments, the tissue was embedded in O.C.T after being dehydrated in 30% sucrose for 24 h at 4 °C. Then, the tissue was snap-frozen with liquid nitrogen and transferred to −80 °C or a cryostat for sectioning at a thickness of 30 μm. The slices and LMMP tissue were washed with 1× PBS 3 times and then incubated overnight with primary antibodies with 0.8% Triton X-100 and 8% goat serum in 1× PBS. For cell immunofluorescence staining, cells were plated on poly-*D*-lysine-coated coverslips in 24-well plates with 500 μL of glial cell media. Cells were fixed with 4% paraformaldehyde for 20 min at room temperature and then washed 3 times with 1× PBS for 7 min each. Next, the cells were incubated with the primary antibody overnight at 4 °C in an antibody dilution buffer containing 2% bovine serum albumin (BSA) and 0.2% Triton X-100. The primary antibodies used were as follows: rat-anti-GFAP (Invitrogen, Cat#13-0300), rabbit-anti-Sox10 (1:200; Abcam, Cat# ab155279), mouse-anti-S100β (1:200; Sigma, Cat# s2532), mouse-anti-TUJ1 (1:1000, Abcam, Cat# ab78078), rabbit-anti-TUJ1 (1:500; Abcam, Cat# ab18207), mouse-anti-HuCD (Invitrogen, Cat# A-21271), rabbit-anti-EDNRB (1:500; Abcam, Cat# ab117529), rabbit-anti-P75 (1:200; HUABIO, Cat# ET1601-22), rabbit-anti-Sox2 (1:200; Abcam. Cat# ab97959), rabbit anti-Synapsin1 (1:500; Abcam, Cat# ab64581), rabbit anti-Calbindin (1:200; Abcam. Cat # ab11426), rabbit anti-Calretinin (1:200; Abcam, Cat# ab16694), rabbit anti-ChAT (1:200; Abcam, Cat# ab18736), rabbit anti-nNOS (1:200; Invitrogen, Cat# PA1-033), and rabbit anti-TH (1:200; Abcam, Cat# ab6211). Cells were incubated with the appropriate secondary antibody for 90 min at room temperature, followed by washing 3 times with 1×PBS. The nucleus was stained with Hoechst 33342 (Sigma). Secondary antibodies included donkey anti-rabbit IgG Alexa Fluor 488 (Invitrogen), goat anti-rat IgG Alexa Fluor 488 (Invitrogen), and goat anti-mouse IgG Alexa Fluor 647 (Invitrogen).

EdU staining strictly referred to the instructions of the BeyoClickTM EdU-488 kit (Beyotime). Images were acquired by an Olympus Fluoview Confocal Microscope or an Olympus IX71 inverted fluorescence microscope.

### Small molecules induce EGC transdifferentiation in vitro

Isolated EGC cells were recovered from liquid nitrogen and seeded as P1 generation cells. The P1 generation cells were passaged again and recorded as P2 cells, which were plated on PDL-coated plates (70,000 cells per well in a 24-well plate or 400,000 cells per well in a 6-well plate). After cultured for 24 h in glial cell medium, the medium was replaced with neuronal induction medium with the indicated chemicals. Neuronal induction medium (NIM) contained DMEM/F12, supplement B-27, N2 (Invitrogen), 20 ng/mL brain-derived neurotrophic factor (BDNF, Invitrogen), and 20 ng/mL glial-derived neurotrophic factor (GDNF, Invitrogen). On Day 4, all of the cultures were replaced with new neuronal induction medium with chemicals.

After 8 days, all cells were cultured in neuronal maturation medium (NMM). During the experiment, we used two different neuronal maturation medium NMM1 (DMEM/F12, supplement B-27, N2, 20 ng/mL bFGF, 20 ng/mL BDNF, 20 ng/mL GDNF) and NMM2 (DMEM/F12, 1% foetal bovine serum, supplement B-27, N2, 10 ng/mL GDNF). The neuronal maturation medium was half-changed every 2–3 days. The chemicals used in the study were as follows: VPA (Selleck, Cat# s1168), CHIR99021 (Selleck, Cat# s1263), RepSox (Selleck, Cat# s7223), EW-7297 (Selleck, Cat# s7530), LY2157299 (Selleck, Cat# s2230), and LDN-193189 (Selleck, Cat# s2618).

### Real-time PCR for detection of target gene expression

Total cellular RNA was harvested using TRIzol (Roche). For reverse transcription, we used 1 μg of RNA to synthesise cDNA by random hexamers and M-MLV reverse transcriptase (Promega) according to the manufacturer’s protocol. Quantitative real-time PCR was performed with primers and 2× SYBR Green qPCR Master Mix (Selleck, Cat# B21702) in an MX3000P Stratagene PCR machine. We used β-actin as an internal control, and relative gene expression was analysed with respect to gene expression at Day 0 for the control. The relevant gene primer sequences we used are shown in Supplementary Table S1.

### Patch-clamp recording

The patch neuronal cells were transferred to the extracellular fluid (126 mM NaCl, 3 mM KCl, 26 mM NaHCO_3_, 1.2 mM NaH_2_PO_4_, 2.4 mM CaCl_2_, 1.3 mM MgCl_2_, 10 mM D-glucose), and the intracellular fluid (93 mM K-gluconate, 16 mM KCl, 2 mM MgCl_2_, 0.2 mM EGTA, 10 mM HEPES, 2.5 mM MgATP, 0.5 mM Na_3_GTP, 10 mM Na-phosphocreatine) was halted in the glass microelectrode. Whole-cell patch-clamp recordings were made with an Axopatch-200B amplifier (Molecular Devices) at room temperature, and data were digitised using a DigiData 1440A with pClamp10.4 software (Molecular Devices). When the whole-cell model was formed after sealing and rupturing, the recording was performed under the condition that the external current was zero. Each neuronal cell spontaneous action potential was recorded for 5 min. In current-clamp mode, we used the current injection from 0.02 to 0.09 nA to record the evoked action potential of neural cells.

### shRNA

The PLL3.7 plasmid was kindly provided by Professor Shao-rong Gao of Tongji University. The shRNA sequences used were shown in Supplementary Table S2. The PLL3.7-ALK5 or PLL3.7-scrambled co-packaging plasmid was transfected into HEK293T cells with Fugene-HD (Roche), and 48 h after transfection, the lentiviral supernatant was collected by filtration through a 0.45 μm filter and centrifuged at 55,000 × *g* for 2.5 h at 16 °C. Then, the supernatant was discarded, and the virus pellet was dissolved in 100 μL of 1× PBS. EGCs were seeded in 6-well plates at a density of 400,000 cells/well or 80,000 cells/well into 24-well plates 1 day earlier. After cultured for 24 h in glial cell medium, cells were infected with the virus suspension containing polybrene (8 μg/mL) and then centrifuged at 1500 rpm for 90 min at 37 °C. The EGC medium was then replaced with neuron induction medium. The efficiency of cells transduction was assessed by the observation of GFP expression under a fluorescence microscope.

### Western blot

Total cellular proteins were obtained by RIPA lysis buffer with protease and phosphatase inhibitors. The protein concentration was calculated by the BCA protein assay kit (Pierce), and 35 μg of protein lysate was loaded onto a 12% SDS-PAGE gel. After electrophoresis, the proteins were transferred to a hydrophobic PVDF membrane (Millipore), which was then blocked by incubation with 5% non-fat dry milk at room temperature for 1 h. The membranes were incubated with the primary antibodies anti-ALK5 (1:1000; Abcam, Cat# ab31013) and anti-GAPDH (Abcam, Cat# ab9485) at 4 °C overnight and washed three times with 1× TBST. After incubation with secondary antibodies, signals were visualised by a chemiluminescence detection system. GAPDH was used as the loading control.

### RepSox induces EGCs transdifferentiation in vivo and gastrointestinal motility studies

Induction of the transformation of EGCs into enteric neurons was performed using 10-week-old adult GFAP-Cre:Rosa26-tdTomato mice in situ. RepSox was dissolved in 0.5% CMC-Na (sodium carboxymethyl cellulose), and the control group was given only 0.5% CMC-Na. RepSox (3 or 10 mg·kg^−1^·d^−1^) and 0.5% CMC-Na were intraperitoneally injected into mice for 2 weeks. The mice were fasted for 20 h with access to water ad libitum. Before the test of intestinal motility, each mouse was given carmine and then free access to water and food ad libitum. The time of the first red stool appearance in the mice was recorded, as well as the number of defecations within 6 h and the wet and dry weight of the feces. After mouse intestinal motility testing, all mice were sacrificed, and the small intestine LMMP tissue was exfoliated to perform immunofluorescence staining.

### EdU^+^ cell labelling in vivo and immunofluorescence co-staining

Seven days after administration of RepSox (3 mg/kg), GFAP-Cre:Rosa26-tdTomato mice were given an intraperitoneal injection of EdU (Beyotime, ST067, 50 mg·kg^−1^·d^−1^). After 14 days of RepSox administration, the mice were sacrificed, the LMMP tissue was stripped and fixed with 4% PFA at 4 °C for 2 h, and then, the tissue cells were stained for EdU according to the procedures of the BeyoClick™ EdU Cell Proliferation Kit with Alexa Fluor 488 (Beyotime, C0071S). For co-staining with HuCD, the LMMP tissue was incubated with HuCD primary antibody overnight and labelled with the corresponding secondary antibody.

### Data statistics

All immunofluorescence staining was statistically analysed using Image-Pro Plus 6.0 software. All graphs were plotted using GraphPad Prism 5 software. Two-tailed Student’s *t* tests were used to calculate statistical significance with *P* values, unless otherwise stated. *P* values < 0.05 (*P* < 0.05) were considered as indicative of significance.

## Results

### Lineage tracing of EGCs in GFAP-Cre:Rosa26-tdTomato mice

**Fig. 1:**
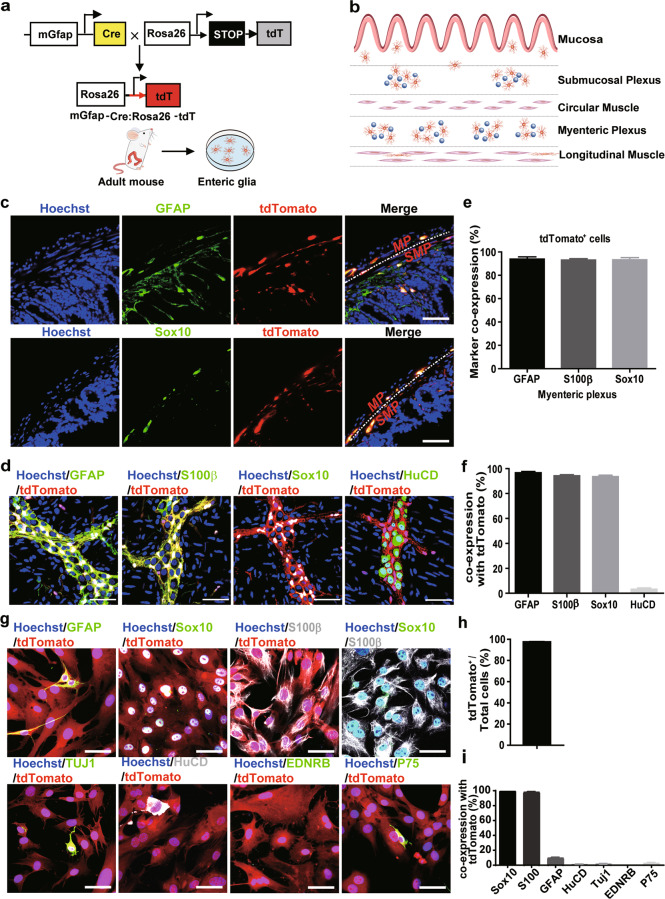
Lineage tracing and identification of EGCs from GFAP-Cre:Rosa26-tdTomato mice. **a** Schematic of EGC lineage tracing approaches using GFAP-Cre:Rosa26-tdTomato mice. **b** Schematic presentation of EGCs distribution in the gut wall in mice. **c** Co-localisation of tdTomato (red) with the EGCs markers GFAP and Sox10 in the small intestine of adult GFAP-Cre:Rosa26-tdTomato mice. Nuclei were labelled with Hoechst 33342 (blue). MP myenteric plexus, SMP submucosal plexus. Scale bar = 50 μm. **d** Immunofluorescence staining of LMMP isolated from adult GFAP-Cre:Rosa26-tdTomato mice. Confocal microscopy showed that tdTomato^+^ cells (red) expressed various enteric glial cell markers GFAP (green), S100β (green), and Sox10 (green). Rather few tdTomoto^+^ cells were labelled by the neuronal marker HuCD (green). Nuclei were stained with Hoechst 33342. Scale bar = 50 μm. **e**, **f** Quantitative analysis of tdTomato^+^ LMMP EGCs in (**d**) (*n* = 3). **e** Percent of cells expressing tdTomato in GFAP^+^, Sox10^+^ or S100β^+^ cells; **f** percent of cells co-stained with GFAP, Sox10 or S100β within the tdTomato^+^ cells. **g** In vitro culture of EGCs. EGCs were isolated from LMMP and characterised by immunofluorescence staining. Nuclei were stained with Hoechst 33342. Scale bar = 50 μm. **h** Quantitative analysis of the tdTomato^+^ EGC percent shown in (**g**) (*n* = 3). **i** Quantitative analysis of cells co-expressing various neuronal markers with tdTomato^+^ in (**g**) (*n* = 3).

Over the last few years, astrocyte-to-neuron conversion in the brain has been intensively studied due to its therapeutic potential in neurodegenerative diseases [11–13, 28, 29]. Direct reprogramming of astrocytes into functional neurons has attracted increasing attention. However, within the ENS, such glia-to-neuron conversion has not yet been well investigated. EGCs also express the astrocyte marker GFAP, and we first tried to use the GFAP gene to trace EGCs in the ENS. GFAP promoter-driven Cre mice were crossed with Rosa26-tdTomato mice harbouring a loxP-flanked STOP cassette preventing the transcription of a DsRed fluorescent protein, tdTomato (Fig. 1a). In offspring mice, Cre recombinase cleaves the STOP cassette, allowing the expression of the downstream tdTomato reporter. Therefore, EGCs and their progeny cells will be labelled and traced with tdTomato fluorescence.

As illustrated in the schematic diagram in Fig. 1b, EGCs are mainly distributed in the myenteric plexus and submucosal plexus. In addition to GFAP, Sox10 and S100β are known as markers of EGCs [24, 30–32]. We performed immunofluorescence staining of GFAP and Sox10 on EGCs within the gut wall of adult GFAP-Cre:Rosa26-tdTomato mice (Fig. 1c). In the cross-section of the gut, red fluorescent cells were observed within the myenteric plexus (MP) and submucosal plexus (SMP) and were colabelled with GFAP and Sox10, which suggests that tdTomato^+^ cells were EGCs. Subsequently, the longitudinal muscle myenteric plexus (LMMP) was isolated from the ileum of adult GFAP-Cre:Rosa26-tdTomato mice and stained with GFAP, Sox10 and S100β (Fig. 1d). The statistical results showed that most GFAP^+^ (93.91% ± 1.90%), Sox10^+^ (93.16% ± 1.05%) or S100β^+^ (93.29% ± 1.81%) cells were tdTomato^+^ (Fig. 1e). In contrast, more than 95% of the tdTomato^+^ cells were co-stained with GFAP (96.73% ± 1.01%), Sox10 (94.26% ± 0.85%) or S100β (93.60% ± 1.12%) (Fig. 1f). In contrast, less than 5% of HuCD^+^ cells (3.27% ± 1.01%) were labelled with tdTomato in the LMMP tissue (Fig. 1f). These results suggest that the tdTomato signal can indicate most of the EGCs in vivo.

Next, EGCs were isolated from LMMP and characterised by immunofluorescence staining in vitro (Fig. 1g). A total of 97.28% ± 0.22% of the isolated cells were tdTomato^+^ (Fig. 1h), and most of them co-expressed Sox10 (100% ± 0.00%) or S100β (98.30% ± 0.20%) (Fig. 1i), suggesting that most of the isolated cells were EGCs. Surprisingly, unlike the staining in vivo, we found relatively little GFAP labelling (9.77% ± 0.47%) in these cells, which might occur due to the highly dynamic expression of GFAP in EGCs, as reported in previous publications [33–37]. TUJ1^+^ tdTomato^+^ (1.70% ± 0.20%) and HuCD^+^ tdTomato^+^ (0.79% ± 0.09%) were mostly absent within these tdTomato^+^ cells (Fig. 1g, i), confirming that very few neurons existed in the isolated EGCs. P75 and EDNRB are general markers for stem cells or neural precursor cells [38, 39]. Immunofluorescence staining showed that 2.70% ± 0.38% of cells were P75^+,^ and no EDNRB^+^ cells were detected (Fig. 1g, i). In summary, our results suggest that the tdTomato^+^ cells in LMMP are mainly EGCs, and tdTomato can be used as a fluorescent tracker for tracing the cell fate of EGCs in vitro and in vivo.

### Induction of neuronal cells from EGCs by the small-molecule compounds VCR and RepSox in vitro

**Fig. 2:**
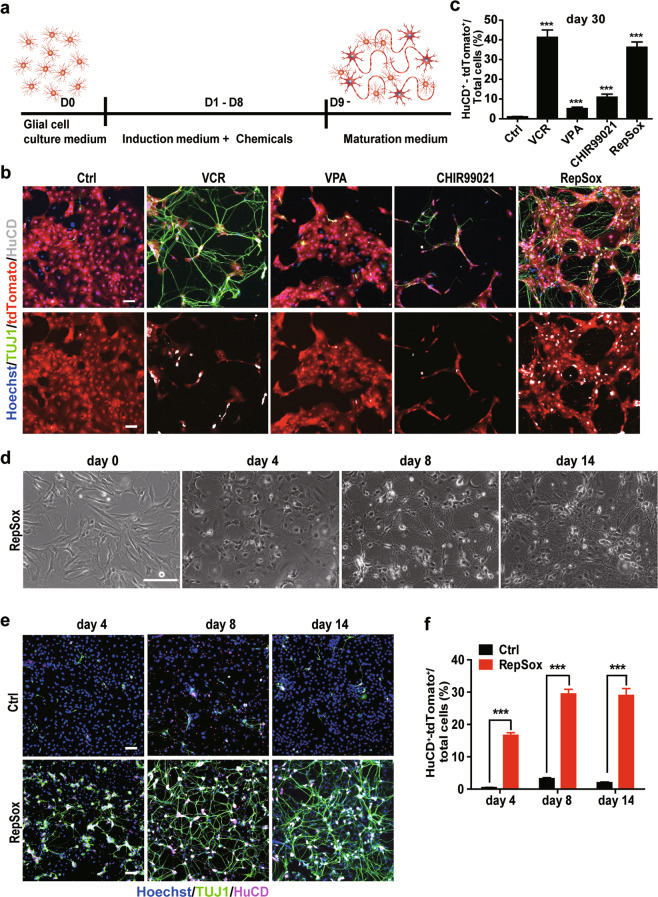
Induction of enteric neurons from EGCs by the small-molecule compound RepSox in vitro. **a** Schematic presentation of the neuronal induction procedure. Initial EGCs were plated in proliferation medium (D0) for 1 day and were transferred into NIM with chemicals for 8 days (D1–D8). Then, the cells were further cultured in NMM for 2–3 weeks. **b** Immunofluorescence staining of the neuron markers TUJ1 (green) and HuCD (pale) to analyse tdTomato^+^ cells treated with chemicals at Day 30. Nuclei were stained with Hoechst 33342 (blue). Scale bar = 120 μm. VCR (V, VPA, 3 mM; C, CHIR99021, 3 μM; R, RepSox, 1 μM); VPA (3 mM); CHIR99021 (3 μM); RepSox (1 μM) **c** quantitative analysis of (**b**). The percent of HuCD^+^-tdTomato^+^ cells among the total cells was analysed at Day 30 after induction (mean ± SEM, *n* = 3, ***P < 0.001). **d** Phase contrast images showing morphological changes of EGCs during neuronal induction on Days 0, 4, 8, and 14. Scale bar = 100 μm. **e** Immunofluorescence staining of TUJ1 (green), HuCD (purple) and DNA (blue) at different time points under RepSox induction. Scale bar = 120 μm. **f** Quantitative analysis of (**e**). The percent of HuCD^+^-tdTomato^+^ cells in total cells at Days 4, 8 and 14 under RepSox induction (mean ± SEM, *n* = 3, ***P < 0.001 vs. Ctrl.).

It has been reported that astrocytes in the central nervous system can be converted into neurons with the small-molecule chemical VCR (including the histone deacetylase inhibitor VPA, the GSK-3 kinase inhibitor CHIR99021 and the TGF-β signalling pathway inhibitor RepSox) [13]. We wondered whether VCR or its single component VPA, CHIR99021, or RepSox could induce transdifferentiation of EGCs into neurons. The experimental procedure is shown in Fig. 2a. After culture in EGCs medium for 24 h, the isolated EGCs were switched to induction medium containing the indicated chemicals for 8 days with medium renewal every 4 days. Then they were further cultured in NMM1 maintenance medium, and half of the culture medium was renewed every 4 days. On Day 30, the cells were fixed with 4% PFA, and immunofluorescence staining was performed (Fig. 2b). Compared to the vehicle control group, VCR treatment led to a dramatic increase in HuCD^+^ cells to 41.25% ± 3.79% of the total cells. Long neurites were extended from these cells, as illustrated by TUJ1 staining (Fig. 2b). Moreover, we found that RepSox alone could also induce EGCs transdifferentiation into enteric neurons with similar efficiency (36.22% ± 2.73%) as VCR. In comparison, only a few neurons were generated under VPA (5.14% ± 0.74%) or CHIR99021 (10.90% ± 1.56%) treatment alone (Fig. 2b, c).

We next analysed the induction efficiency of RepSox at different time points according to the procedure shown in Fig. 2a and investigated the morphological changes of EGCs. Upon RepSox treatment (added to the induction medium from Day 1 to Day 8), EGCs gradually changed their shape and grew neuron-like axons or dendrites over time (Fig. 2d). Cells were subjected to TUJ1 and HuCD double immunofluorescence staining (Fig. 2e). The proportion of HuCD^+^tdTomato^+^ cells was approximately 16.65% ± 0.79% on Day 4 of RepSox induction, reached 29.44% ± 1.46% on Day 8, and maintained a plateau on Day 14 (28.95% ± 0.38%) (Fig. 2f). Immunofluorescence staining of TUJ1 showed that cells grew longer and denser neurites within the extended culture (Fig. 2e). These results suggest that EGCs can be sufficiently converted into enteric neurons by RepSox in vitro.

### RepSox can induce the transdifferentiation of EGCs into neurons without reprogramming EGCs to neural stem cells in vitro

**Fig. 3:**
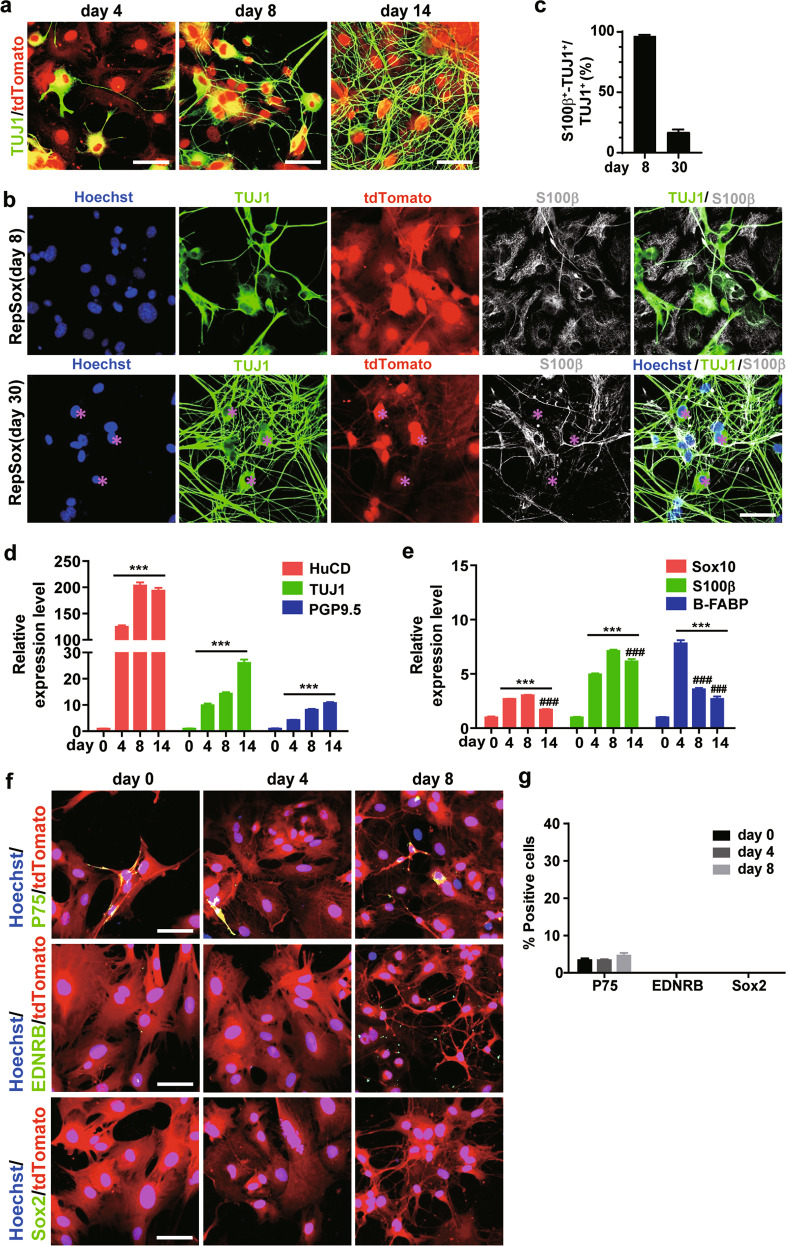
RepSox can induce the transformation of EGCs into neurons without reprogramming of EGCs to neural stem cells in vitro. **a** TUJ1 immunofluorescence staining was performed on Days 4, 8 and 14 of culture to reveal the morphological changes of the cells. Scale bar = 50 μm. **b** Immunofluorescence staining analysis of S100β and TUJ1 on Days 8 and 30 after RepSox induction, respectively. Scale bar = 50 μm. **c** Statistics of (**b**) (mean ± SEM, *n* = 3). **d**, **e** Real-time PCR analysis of mRNA expression in cells during RepSox induction. Data were shown as the fold change versus Day 0 (mean ± SEM, *n* = 3, ***P < 0.001 vs. Day 0; ^###^*P* < 0.001 vs. its corresponding maximum expression). **f** Immunofluorescence staining was performed to analyse the expression of the neural stem cell markers P75, EDNRB and Sox2 in EGCs 0, 4 and 8 days after RepSox treatment. Scale bar = 50 μm. **g** Statistical analysis of (**f**).

To further monitor the detailed process of EGC-neuron conversion, we performed immunofluorescence staining of S100β together with TUJ1. The proportion of TUJ1^+^tdTomato^+^ cells was significantly increased with prolonged culture time (Fig. 3a, b). Co-staining of S100β revealed that the S100β immunofluorescence signal gradually decreased and eventually diminished in some TUJ1^+^ cells (labelled in Fig. 3b with asterisks). Statistical analysis showed that the proportion of TUJ1^+^ cells expressing S100β decreased from 96.74% ± 0.85% on Day 8 to 16.92% ± 2.29% on Day 30, suggesting the transdifferentiation of EGCs into neurons (Fig. 3b, c). During RepSox treatment, neuron- or EGC-specific gene expression was tested by qPCR (Fig. 3d, e). The mRNA levels of the neuronal markers HuCD, TUJ1, and PGP9.5 increased persistently upon the addition of RepSox. In parallel, the mRNA levels of the glial markers Sox10, S100β, and B-FABP increased initially but dropped dramatically on Day 14. These gene expression profiles are consistent with the glia-to-neuron switch triggered by RepSox.

Next, immunofluorescence staining of the neural stem cell or neural precursor cell markers P75, EDNRB, and Sox2 were carried out. The results showed that very few cells in the original cell population expressing P75^+^ remained unchanged during RepSox induction (Day 0, 3.38% ± 0.46%; Day 4, 3.37% ± 0.74%; Day 8 4.60% ± 0.72%). In addition, no EDNRB^+^ or Sox2^+^ cells appeared, excluding the possibility that RepSox-induced neurons were derived from stem cells in the ENS (Fig. 3f, g). These results suggest that EGCs can be sufficiently converted into enteric neurons by RepSox without reprogramming to neural stem cells in vitro.

### RepSox-induced EGC-derived neurons have electrophysiological activity and are mainly Calbindin- and nNOS-positive neurons

**Fig. 4:**
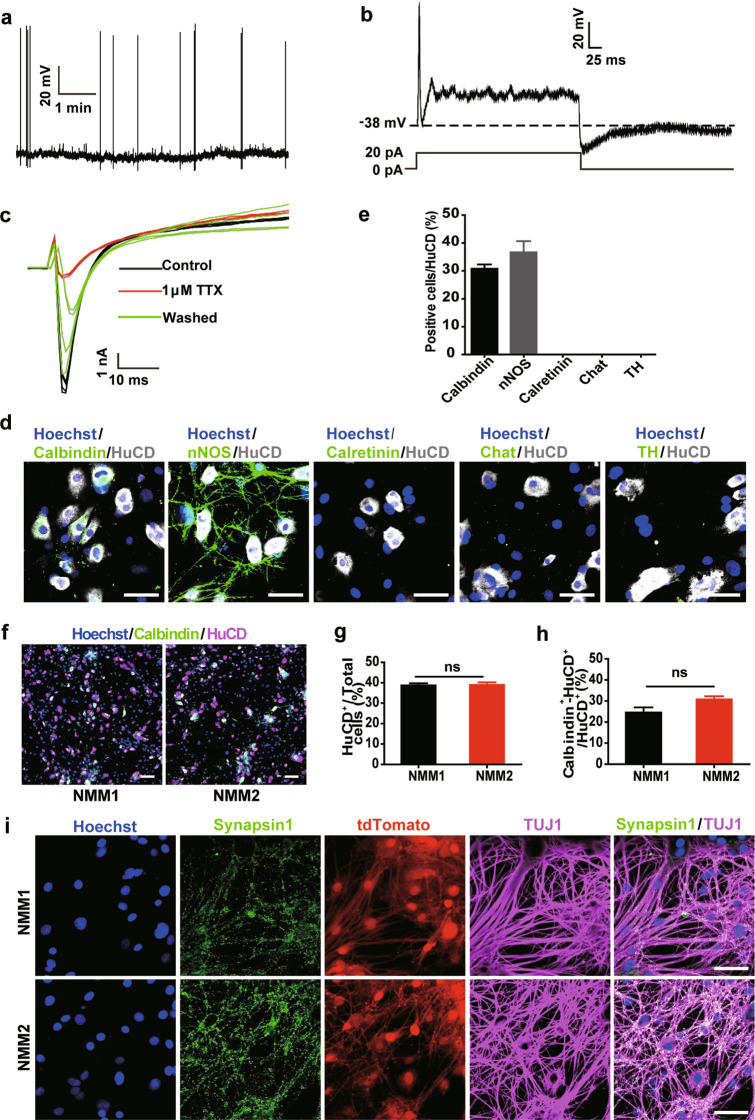
RepSox-induced EGCs-derived neurons had electrophysiological functions and expressed Calbindin and nNOS in vitro. **a** The induced neurons had spontaneous action potentials at Day 19 (*n* = 12). **b** In current-clamp mode, the induced neurons displayed action potentials at a current injection of 0.02 nA. Upon cessation of current stimulation, neurons returned to their original resting membrane potential (*n* = 8). **c** Tetrodotoxin (TTX)-sensitive and TTX-resistant Na^+^ channels were present in neurons. Raw tracing showed that 1 μM TTX caused a decrease in the Na^+^ current (*n* = 4). **d** Immunofluorescence staining of induced neurons with different enteric neuronal markers Calbindin, Calretinin, nNOS, Chat, and TH. Scale bar = 50 μm. **e** Quantitative analysis of (**d**) revealed that Calbindin- and nNOS-positive neurons were the main cell population upon RepSox induction (*n* = 3). **f** Cells were cultured in NMM1 and NMM2 for 19 days and stained with Calbindin and HuCD antibodies. Scale bar = 120 μm. **g**, **h** Statistical analysis of (**f**) (mean ± SEM, *n* = 3). **i** Synapsin1 immunofluorescence staining after induction of EGCs by RepSox. More Synapsin1 protein (green) was produced when culturing in NMM2 medium for 19 days. Scale bar = 50 μm.

Next, we investigated whether EGC-derived neuronal cells had functional maturity. To promote neuron maturation, EGC-derived neurons were cultured in 1% foetal bovine serum (FBS)-containing maintenance medium (NMM2), a condition that has been reported to improve the spontaneous action potentials in enteric neurons [40]. Serum-free medium (NMM1) was also tested. Using the whole-cell patch-clamp recording technique, we found that on Day 19 after RepSox (1 μM) treatment, the converted neurons were able to show spontaneous action potentials when maintained in NMM2 (Fig. 4a); however, until Day 70 after RepSox treatment, no spontaneous electrophysiological activity could be detected when the converted cells were cultured in NMM1. In current-clamp mode, all enteric neurons exhibited action potentials at current injections ranging from 0.02 to 0.09 nA. At the end of the current pulse, the enteric neurons returned to their original resting membrane potential (Fig. 4b). In addition, tetrodotoxin (TTX), a selective inhibitor of Na^+^ channels, significantly inhibited Na^+^ inward currents (Fig. 4c). These results suggested that RepSox-induced EGC-derived neurons were electrophysiologically mature.

Many types of neurons exist in the myenteric plexus of mice, and many neurodegenerative diseases are caused by the damaging of a specific type of neurons [41–43]. We tried to analyse the subtype of the obtained neurons in our transdifferentiation system. Immunofluorescence staining showed that ~30.77% ± 1.54% of the converted neurons were Calbindin positive and 36.66% ±3.97% were positive for nNOS (Fig. 4d). No cells were positive for Calretinin, TH or Chat (Fig. 4d, e), indicating that the converted neurons were mainly responsible for gut muscle relaxation and gut motility control. In addition, we compared the EGC-neuron conversion efficiency induced by RepSox under different NMM culture conditions. Staining of HuCD and Calbindin on Day 19 revealed that the number and subtype of neurons produced were nearly the same under both NMM culture conditions (Fig. 4f–h). However, the neurons cultured in NMM2 medium had more synapsin1 expression and denser dendritic branches than those cultured in NMM1 medium, as shown by TUJ1 and synapsin1 staining on Day 19 (Fig. 4i), suggesting that the addition of FBS can indeed make enteric neurons more mature.

### TGFβR-1/ALK5 signalling pathway plays a crucial role in the transdifferentiation of EGCs to enteric neurons

**Fig. 5:**
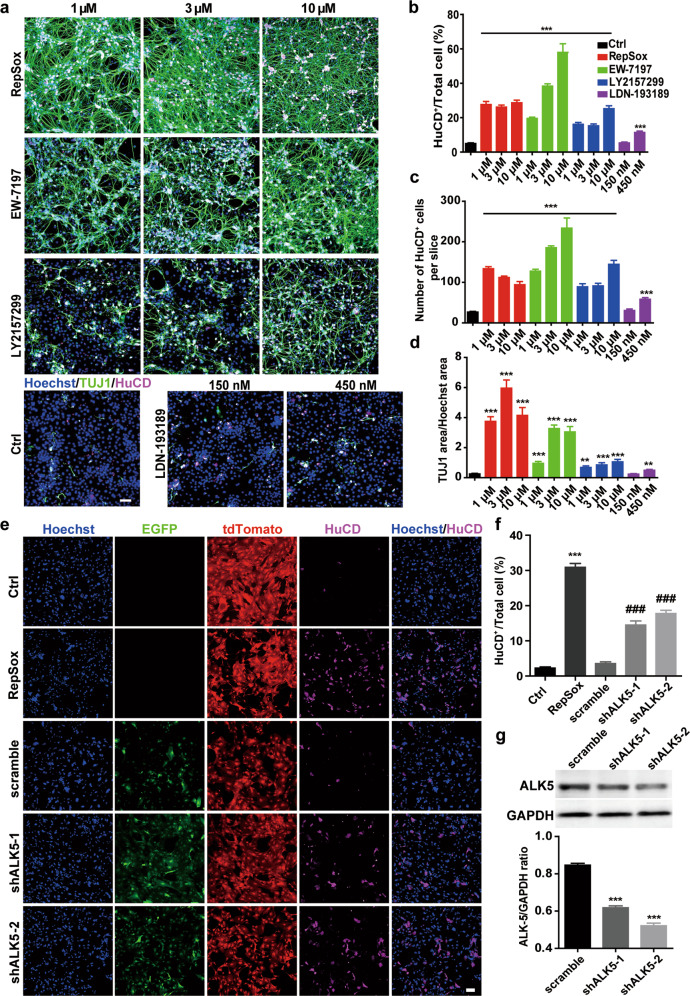
Inhibition of the TGFβRI/ALK5 signalling pathway contributed to the EGCs-neuron transdifferentiation. **a** Immunofluorescence staining of HuCD and TUJ1 in cells after treatment with selective inhibitors of ALK5, RepSox, EW-7197 and LY2157299 (1, 3, 10); a non-Alk5 inhibitor, but a selective BMP type I receptor kinase inhibitor, LDN-193189, was also tested. Scale bar = 120 μm. **b**–**d** Quantitative analysis of (**a**). Percentage of HuCD^+^ neurons in the total number of cells (**b**), number of HuCD^+^ neurons per slide (**c**), ratio of TUJ1 area to Hoechst 33342 area (**d**) (mean ± SEM, *n* = 3, **P < 0.01; ***P < 0.001 vs. Ctrl.). **e** Immunofluorescence staining of HuCD after ALK5 knockdown in EGCs. Scale bar = 120 μm. **f** Statistics of (**e**). The percent of HuCD^+^ neurons in the total number of cells (mean ± SEM, *n* = 3, ***P < 0.001 vs. Ctrl; ^###^*P* < 0.001 vs. scramble). **g** Western blot showed the knockdown efficiency of the two ALK5-shRNAs in EGCs (mean ± SEM, *n* = 3, ***P < 0.001 vs. scramble).

RepSox is a selective inhibitor of TGFβR-1/ALK5 that was used to replace Sox2 and c-Myc in the iPSC induction system by blocking the TGF-β signalling pathway and inducing Nanog gene expression [44]. Our previous results have revealed that RepSox can efficiently transdifferentiate EGCs into enteric neurons in vitro, indicating that TGFβR-1/ALK5 signalling might be the critical pathway in this process. To clarify this possibility, we chose several other selective inhibitors of TGFβR-1/ALK5 and investigated their effects on EGC-neuron conversion. EW-7197 and LY2157299 are two other known TGFβR-1/ALK5 selective inhibitors, while LDN-193189 (DM3189) is a selective BMP signal inhibitor for ALK1, ALK2, ALK3, and ALK6. Interestingly, EGC-neuron conversion can be achieved by EW-7197 and LY2157299 in a concentration-dependent manner similar to RepSox (Fig. 5a–d). In contrast, LDN-193189 was less efficient and already exhibited a cytotoxic effect at 1 μM. The results suggest that the TGFβR-1/ALK5 signalling pathway plays a more critical role in the EGC-neuron conversion process.

Next, we utilised shRNA to downregulate ALK5 expression in EGCs. Two shRNAs, namely, shALK5-1 and shALK5-2, and one scrambled shRNA was introduced into EGCs. Western blot analysis revealed ~50% downregulation of ALK5 protein levels in EGCs (Fig. 5g). Immunofluorescence staining of HuCD at Day 20 showed that knockdown of ALK5 could also successfully promote transdifferentiation of EGCs into enteric neurons (Fig. 5e, f). Compared to the RepSox (1 μM) treatment, the compromised transdifferentiation efficiency might be explained by the only 50% downregulation of ALK5. In conclusion, selective inhibition of TGFβR-1/ALK5 or reduction of ALK5 expression can promote the transdifferentiation of EGCs into enteric neurons, demonstrating that the TGFβR1/ALK5 signalling pathway indeed plays an essential role in EGC-neuron transdifferentiation.

### Daily RepSox gavage promotes the conversion of EGCs into neurons and influences gastrointestinal motility in mice

**Fig. 6:**
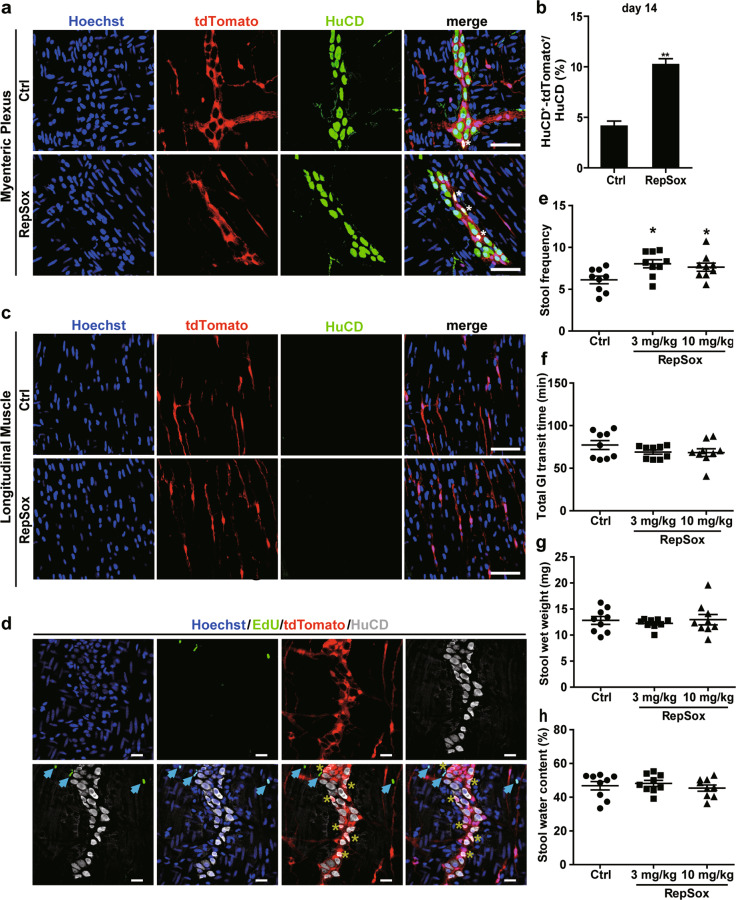
Daily RepSox gavage can promote the conversion of EGCs into neurons and improve GI motility in vivo. **a** Immunofluorescence staining for HuCD (green) in the myenteric plexus of tdTomato mice treated with RepSox (3 mg·kg^−1^·d^−1^) for 14 days. Scale bar = 50 μm. **b** Quantitative analysis of (**a**) showed the proportion of HuCD^+^-tdTomato^+^ cells in total neuronal cells (HuCD^+^) in ganglia (*n* = 10, **P < 0.01). **c** After 14 days of treatment with RepSox in vivo, HuCD immunofluorescence staining showed no HuCD^+^-tdTomato^+^ cells appearing in longitudinal muscles. Nuclei were labelled with Hoechst 333422 (blue). Scale bar = 50 μm. **d** In the second week with RepSox treatment, mice were injected with EdU intraperitoneally for 7 days. LMMPs were co-stained with HuCD (pale) and EdU (green). Nuclei were labelled with Hoechst 33342 (blue). Scale bar = 20 μm. **e**–**h** GI motility test in vivo. Stool frequency (**e**); GI transit time (**f**); Stool wet weight (**g**); Stool water content (**h**). (*n* = 9 for each group, *P < 0.05, **P < 0.01 vs. Ctrl.).

Since our in vitro experiments have demonstrated that EGCs from the myenteric plexus have a high potential of transdifferentiation into enteric neurons after induction with RepSox, we wondered whether RepSox can induce EGC transition into enteric neurons in vivo. We tested this possibility by daily intragastric administration of RepSox to GFAP-Cre:Rosa26-tdTomato mice for 2 weeks and tracing the cells by tdTomato fluorescence. Mouse LMMP tissues were isolated, and HuCD immunofluorescence staining showed that 3 mg/kg RepSox administration doubled the proportion of HuCD^+^ tdTomato^+^ cells in ganglia compared with that in the vehicle control-treated mice (Fig. 6a, b). There are four types of mouse EGCs. Type I-III EGCs belong to intraganglionic glial cells, which are located adjacent to the enteric nerve cells in the nerve tubercle within the submucosal plexus and myenteric plexus layer of the intestine (see Fig. 1b). The type IV EGCs are located in the longitudinal muscle region, where no nerve tubercles exist. Interestingly, we found that RepSox did not convert IV EGCs located inside the longitudinal muscle (Fig. 6c). In addition, we performed an EdU incorporation assay to analyse cell proliferation within the myenteric plexus. Only a few EdU-positive cells were detected, mainly exclusive from either tdTomato- or HuCD-stained cells. These results suggest that RepSox treatment did not alter cell proliferation within the myenteric plexus, and the increased number of HuCD^+^tdTomato^+^ cells induced by RepSox in vivo was derived directly from EGCs without significant cell expansion (Fig. 6d), unlike enteric neural stem cells.

Two weeks after RepSox administration (3 mg/kg or 10 mg/kg), gastrointestinal motility was measured in mice. The stool frequency was significantly increased, but the intestinal transit time was not changed compared with that of the control group (Fig. 6e, f). The wet weight and water content of stool were not significantly different from those of the control group (Fig. 6g, h).

In summary, our work suggests that RepSox can increase the number of enteric neurons in a manner consistent with transdifferentiation from EGCs. These findings might provide therapeutic developments for the treatment of enteric neuropathies with neuron loss.

## Discussion

Intestinal diseases are often associated with neuronal death that could, in turn, compromise intestinal functionality [45, 46]. Enteric neurogenesis and regeneration is therefore a crucial aspect for the recovery of ENS neuropathy. In fact, accumulating evidence supports ENS neuron regeneration after birth and in adult individuals [27]. However, the incidence of overall ENS neurogenesis remains very low in adulthood, and the underlying mechanisms remain elusive. In the current study, we demonstrate that a TGFβR-1/ALK5 inhibitor, RepSox alone, can induce a sufficient amount of EGC conversion into enteric neurons in vitro and in vivo. Furthermore, knockdown of ALK5 resulted in an effect similar to that of RepSox, confirming that the TGFβR-1/ALK5 signalling pathway plays a crucial role in the transdifferentiation of EGCs to enteric neurons. Our findings revealed an essential pathway regulating the glia-to-neuron switch in the adult ENS, which might be considered a promising target for developing therapeutic strategies to repair ENS neuron damage in various neurodegenerative diseases.

Although it has become clear that neurogenesis can occur in the adult ENS, the circumstances that provoke it and the underlying mechanism is largely unknown. Several mouse models have been used to analyse enteric neuronal development in vivo at the adult stage [45], and certain nonpathological or pathological conditions affecting ENS neurogenesis have been discovered. For instance, it was shown that the postnatal/adult development of ENS neurons is 5-hydroxytryptamine (5-HT; serotonin) and 5-HT receptor 4 (5-HT_4_R) dependent [26, 47]. The 5-HT_4_ agonist was able to promote stem cells to proliferate outside the myenteric plexus in the intestinal muscle and to migrate towards the ganglia. In a murine dextran sulphate sodium (DSS) colitis model, myenteric neurons were derived from nestin cells in a 5-HT_4_R-dependent manner [48]. Another study demonstrated that LY3201, a selective ERβ agonist, was able to stimulate glia-to-neuron cell differentiation in vitro and promoted neurogenesis in two murine models of enteric neuronal damage and loss, namely, high-fat diet and the cationic detergent benzalkonium chloride (BAC) injury on the intestinal serosa [49]. Other signalling molecules, such as phosphatase and tensin homologue (PTEN), were also reported to be involved in regulating precursor proliferation and the number and size of neurons in the myenteric plexus [50]. Recently, the unique roles of the intestinal microbiota in postnatal and adult neurogenesis were revealed, as its molecular determinants, lipopolysaccharide (LPS) and short-chain fatty acids (SCFAs), can regulate enteric neuronal survival and stimulate neurogenesis [51]. Previous studies in the CNS have demonstrated that the small-molecule compounds VCR and VCRFBI (VCR, forskolin, i-Bet151, and ISX-9) can directly convert mouse or human adult brain astrocytes into neurons in vitro, respectively [12, 13]. However, these compounds and their related signalling pathways have never been assessed in the ENS. Interestingly, when we tested these compounds in the ENS, we observed similar glia-to-neuron induction by VCR in the ENS as in the brain. Surprisingly, a single chemical component of VCR, RepSox, can stimulate efficient ENS neurogenesis in cultured EGCs and in adult mice under physiological conditions by oral administration. RepSox inhibits the binding of ATP to ALK5, inhibits ALK5 autophosphorylation [52] and can successfully displace Sox2 by inhibiting transforming growth factor-β (TGF-β) signalling in reprogramming, which in turn leads to Nanog expression [44]. EW-7197 is a potent, selective, orally bioeffective TGFβ receptor ALK4/ALK5 inhibitor that can inhibit TGFβ-induced Smad2 or Smad3 phosphorylation and epithelial to mesenchymal transition (EMT) in TGFβ-treated breast cancer cells [53]. LY2157299 is a potent TGFβ receptor inhibitor that inhibits TGFβ-mediated Smad2 activation and haematopoietic suppression in a dose-dependent manner in primary haematopoietic stem cells [54]. Our study found that all these compounds had similar effects as RepSox in promoting the transdifferentiation of EGCs into enteric neurons. TGFβR-1/ALK5 inhibitors have been used as essential components of small-molecule cocktails to induce direct conversion of fibroblasts into cardiomyocytes [55], neural cells [56, 57], osteoblasts [58], and adipogenesis [59]. Therefore, TGFβR-1/ALK5 signalling plays an essential role in achieving certain types of cell fate conversion. Bone morphogenetic proteins (BMP) and TGFβ share similar receptors and messengers. When testing LDN-193189 (DM3189), a selective BMP signal inhibitor for ALK1, ALK2, ALK3, and ALK6, we observed relatively inefficient glia-to-neuron induction. BMP is involved in cell cycle exit of enteric neural crest cells to govern neural differentiation during ENS development [27]. Their roles remain contradictory since they participate in both gliogenesis and neurogenesis. Information concerning the role of BMP in the adult ENS is rather limited. Whether the inefficient induction with BMP inhibition in our study is due to the developmental stage needs to be further investigated.

In addition to the complicated mechanisms underlying adult ENS neurogenesis and gliogenesis, the origin of differentiating cells is always under debate. Putative candidates are an embryonic-like enteric neural progenitor population, Schwann cell precursors and transdifferentiating glial cells. Enteric neural crest-derived cells, such as neural progenitors, still exist in the intestine and might continue supplying neuronal cells in the ENS under physiological and pathophysiological conditions postnatally [24, 26, 60]. There are also several evidences showing that under certain conditions, EGCs have the ability to proceed with enteric neurogenesis in vitro and in vivo [24–26, 50, 61]. Using GFAP-Cre:Rosa26-tdTomato mice, we were able to trace the converted cell origin by tdTomato fluorescence. With detailed characterisation, we concluded that the tdTomato-positive cells in our study were mostly EGCs or their progeny. Furthermore, RepSox-induced neurons were tdTomato^+^, supporting that they were from EGCs. Consistently, the EdU incorporation assay also ruled out the possibility that the converted cells were embryonic-like enteric neural progenitors. Regarding the reprogramming process, our results suggest a direct conversion from EGCs to enteric neurons, since no stem markers were detected during the conversion process, which is in agreement with a previous report demonstrating a direct astrocyte-to-neuron conversion by VCR in the brain [13]. RepSox-induced enteric neurons were characterised as Calbindin^+^ and nNOS^+^ neurons with mature electrophysiological activity and can alter gut motility in mice. When we treated normal GFAP-Cre:Rosa26-tdTomato mice with RepSox by gavage for 2 weeks, we found that RepSox promoted the conversion of EGCs into enteric neurons in myenteric plexus ganglia. However, EGCs in longitudinal muscles were not transdifferentiated into enteric neurons. These results suggest that different types of EGCs may play different roles in maintaining gut homoeostasis and have different plasticity and functions.

So far, our studies were performed in normal mice. How RepSox and TGFβ signalling act under pathological conditions in the gut needs to be further investigated. Small-molecule chemicals are easy to manipulate and flexible to combine, and their biological effects are usually reversible and finely adjustable. More importantly, they can be administered orally. RepSox gavage can promote the in situ conversion of EGCs into enteric neurons, thereby offering a convenient means to compensate for enteric neuronal loss during gastroenterological diseases.

Taken together, our results provide evidence for the essential role of TGFβ signalling in adult ENS neurogenesis, which can be considered an enticing therapeutic strategy for gastrointestinal neuropathy.

## Supplementary information

Supplementary Table S1

Supplementary Table S2
